# Polymorphism rs2682818 participates in the progression of colorectal carcinoma via miR-618-TIMP1 regulatory axis

**DOI:** 10.1038/s41598-021-02613-4

**Published:** 2021-11-30

**Authors:** Wei Shao, Haina Xia, Qiangfang Lan, Jialu Gu, Haidong Huang, Fei Zheng, Youyou Zheng

**Affiliations:** Zhoushan Putuo District People’s Hospital, Zhoushan, 316100 China

**Keywords:** Colorectal cancer, Cell death, Cell division, Cell migration, Cell signalling

## Abstract

Colorectal carcinoma (CRC) has a high morbidity and mortality. Current studies have confirmed a variety of microRNA polymorphisms were associated with tumor susceptibility, however, the mechanisms are still unknown. In this study, we were aimed to clarify how polymorphism rs2682818 participated in the progression of CRC. First of all, the differential expression of miR-618 was assessed by quantitative real-time polymerase chain reaction in CRC patients with different genotypes of polymorphism rs2682818, including homozygous (TT) genotype, homozygous (GG) genotype and heterozygous (TG) genotype. Secondly, plasmids carried miR-168 precursor sequences harboring rs2682818 (SNP type) or without rs2682818 (wild type) were transfected into 293T cells to verify that polymorphism rs2682818 affected miR-618 expression. Thirdly, CCK-8 assay, flow cytometry assay, transwell assay and mouse xenograft assay were performed to measure the biological functions of miR-618 in CRC. Fourthly, the candidate target genes of miR-618 which were predicted by bioinformatics tools were verified by luciferase reporter assay. Finally, in order to explain the potential molecular mechanisms, western blotting was performed to demonstrate the differential expression and phosphorylation of pathway related proteins. The results showed that miR-618 was down-regulated in colon cancer, especially in CRC patients with rs2682818 GG homozygous genotype. Higher expression of mature miR-618 occurred in patients with TT homozygous genotype, and these patients usually had a longer survival time. Moreover, miR-618 mimic obviously impaired the growth and invasion ability of CRC cells, and miR-618 mimic also remarkably promoted CRC cell apoptosis. Our luciferase experiments confirmed that TIMP1 was a target of miR-618 in CRC cells. Knockdown of TIMP1 also significantly inhibited the malignant cytological features of CRC, including malignant growth and invasion as well as apoptosis resistance. In summary, polymorphism rs2682818 participated in the progression of CRC via affecting the expression of mature miR-618 in CRC cells, and miR-618 inhibited the progression of CRC via targeting TIMP1expression.

## Introduction

Colorectal carcinoma (CRC) is one of the most common solid tumors in the worldwide^1^. Studies have confirmed that many risk factors are related to the occurrence and progression of CRC, including germline genetic mutations^2^, associated diseases^3^, environmental exposures^4^, lifestyle and dietary factors^5^. In the past decade, studies have revealed that single nucleotide polymorphisms (SNPs) are associated with CRC risk, including those SNPs in microRNAs^6–8^.

MicroRNAs are small noncoding RNAs, which play an important role in gene expression^9^. Numerous studies have confirmed that microRNAs participate in cancer development and progression, functioning as oncogenes and tumor suppressor genes via interfering with expression of downstream key genes^10–12^. SNP (Single nucleotide polymorphism) is the substitution of a single nucleotide at a special position in the genome, in which each variation exists to a certain extent in the population. SNPs may occur in the coding sequence, non-coding region or intergenic region of genes. Most SNPs have no effect on health or development. However, some SNPs have been proved to be very important in human health research. Researchers have found that SNP may be helpful to predict the individual's response to certain drugs, susceptibility to environmental factors such as toxins and risk of specific diseases. SNP can also be used to trace the inheritance of disease genes in families. Future research will help identify SNPs associated with complex diseases, such as heart disease, diabetes and cancer^13–15^. In the past decades, more and more SNPs in microRNAs or pre-microRNA genes have been reported to be associated with various kind of human cancer, nevertheless, how do these SNPs work is still need to be further study^16–18^.

Recent studies have found that the abnormal expression of miR-618 is related to tumorigenesis^19–21^. Rs2682818 polymorphism is located on the precursor’s stem-loop of the miR-618 sequence and several studies have demonstrated that rs2682818 is associated with diseases susceptibility, including ischemic stroke and hirschsprung disease^22,23^. In addition, a few studies also believe that rs2682818 is related to cancer susceptibility, including colorectal cancer and breast cancer^24,25^. However, none of the above studies can provide enough convincing evidence to explain the correlation between rs2682818 and tumorigenesis. Therefore, it is a very attractive and significant scientific problem to confirm the relationship between rs2682818 polymorphism of miR-618 and colorectal carcinoma susceptibility and explain how is rs2682818 polymorphism involved in the occurrence and progression of colorectal carcinoma.

In this study, we found that polymorphism rs2682818 was associated with CRC risk, and the survival of CRC patients with rs2682818 TT homozygous allele were significantly better than those patients with TG heterozygous genotype or GG homozygous genotype. Moreover, the expression level of matured miR-618 is remarkably higher in lovo cell line (TT homozygous genotype) than SW480 (TG heterozygous genotype) and HT29 (GG homozygous genotype). On this basis, we investigated the relationship between polymorphism rs2682818 and the expression of miR-618, and furtherly clarified the effects of miR-618 in CRC and the underlying mechanism.

## Results

### Polymorphism rs2682818 is associated with the risk of CRC

**Figure 1:**
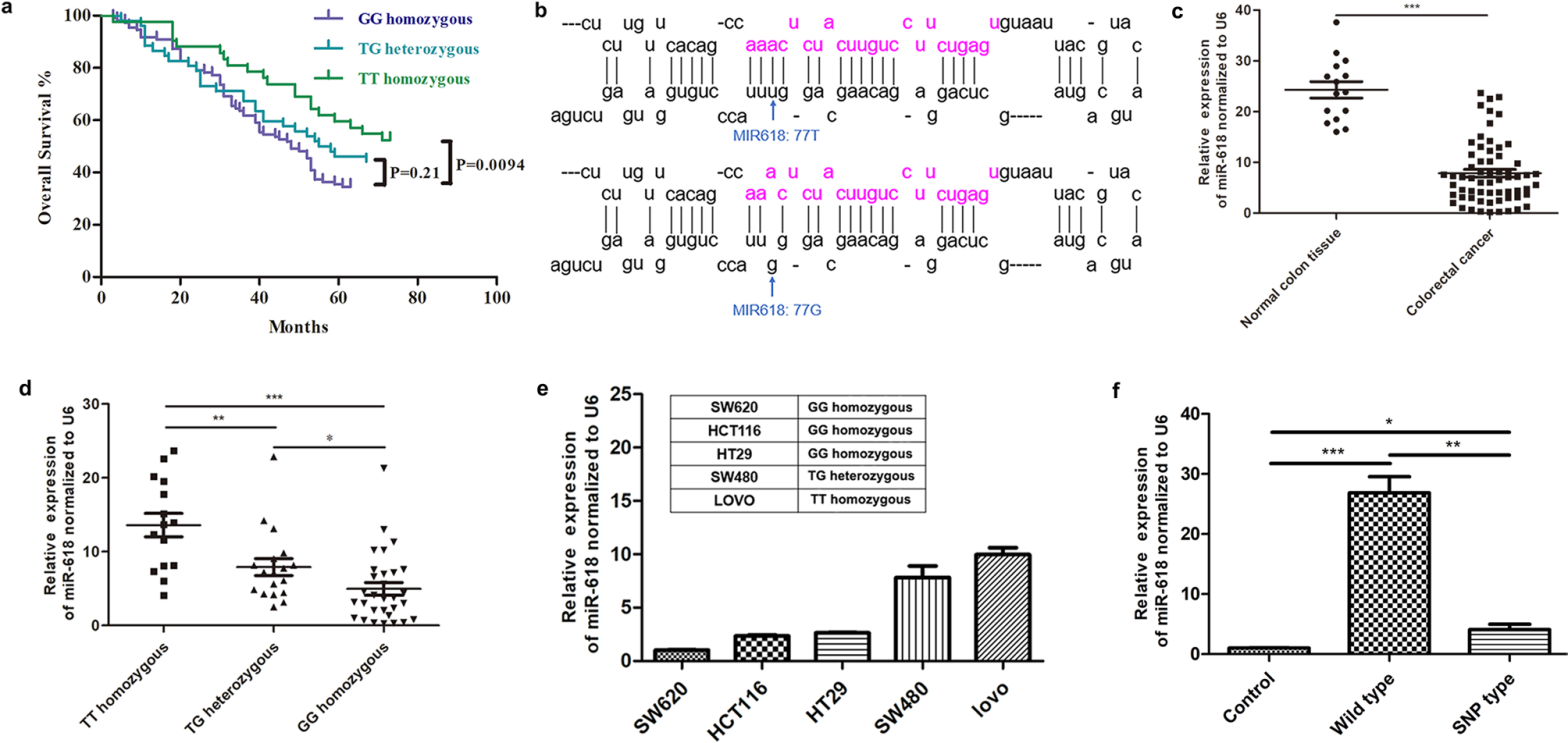
Polymorphism rs2682818 is associated with down-regulation of miR-618 and the CRC risk. (**a**) Kaplan–Meier survival curve constructed for totally 205 colorectal cancer patients, who were divided into three groups according to the rs2682818 allele: homozygous (TT) genotype (n = 111) , homozygous (TG) genotype (n = 52) and homozygous (GG) genotype (n = 42). The survival data of each group were used to construct the K-M survival curve with CI = 95%, which can be further used as the historical reference survival curve. (**b**) In silico prediction of the secondary structure of different pre-miR-618 molecules. (**c**) q-PCR analysis of miR-618 in normal colon tissues and colorectal cancer tissues, in which U6 served as an internal control. Statistical analysis is done for groups with line, and the adjusted *p* value is represented above the line. ***P < 0.001 (t-test). (**d**) q-PCR analysis of miR-618 in colorectal cancer tissues which were divided into three groups, and U6 served as an internal control. Statistical analyses are done for groups with lines, and the adjusted *p* value are represented above the lines. *P < 0.05, **P < 0.01, ***P < 0.001 (t-test). (**e**) q-PCR analysis of miR-618 in different colorectal cancer cell lines, and U6 served as an internal control. (**f**) q-PCR analysis of miR-618 in 293T cells transfected with two kind of miR-618 expression plasmids, which were separately carried miR-168 precursor sequences harboring rs2682818 (SNP type) or without rs2682818 (wild type). *P < 0.05, **P < 0.01, ***P < 0.001 (t-test).

To explore the impact of polymorphism rs2682818 on CRC risk, we analyzed the nucleotide variation characteristics of all the studied subjects. As shown in Table 1, the positive rate of rs2682818 GG homozygous in CRC patients is significantly higher than that in colitis patients. And next, survival analysis were performed to assess the impact of polymorphism rs2682818 in CRC patients, our result revealed that patients with GG homozygous or TG heterozygous genotype had a worse overall survival compared with those patients with TT homozygous genotype (Fig. 1a).

**Table 1:** Nucleotide variation characteristics of studied subjects.

Group	rs2682818 genotype, n (%)						*P*-value^1^		
	Homozygote TT		Heterozygote TG		Homozygote GG				
	Positive	Negative	Positive	Negative	Positive	Negative			
Colitis patients (n = 39)	21 (53.85)	18 (46.15)	11 (28.21)	28 (71.79)	7 (17.95)	32 (82.05)			
Colorectal cancer patients (n = 205)	52 (25.37)	153 (74.63)	42 (20.49)	163 (79.51)	111 (54.15)	94 (45.85)	0.0003	0.284	3.38E-05

^1^Two-sided χ^2^ test for the distributions of homozygote TT (1st column), heterozygote TG (2nd column) and homozygote GG (3rd column) between colorectal cancer patients and Colitis patients as controls.

### Polymorphism rs2682818 is associated with the expression of miR-618 in CRC tissues

As shown in Fig. 1b, we predicted the secondary structure of different pre-miR-618 molecules harboring rs2682818 (SNP type) or without rs2682818 (wild type) in silico (Fig. 1b), we speculated that polymorphism rs2682818 would interfere the cleavage of pre-miR-618. So we detected the expression of mature miR-618 in normal colon tissues, CRC tissues and cell lines with different rs2682818 genotype. The results revealed that the expression of mature miR-618 in CRC tissues was significantly lower than that in normal colon tissues (Fig. 1c). And the expression of mature miR-618 in CRC tissues or CRC cell lines with GG homozygous and TG heterozygous genotype was notably lower than those TT homozygous controls (Fig. 1d,e). In order to investigate the effect of polymorphism rs2682818 on the maturation of miR-618, we constructed two miR-618 expression plasmids separately harboring rs2682818 or without rs2682818 in the miR-168 precursor sequence. The expression of mature miR-618 was detected by qPCR in 293T cells which were respectively transfected with these two plasmids. Our result revealed that rs2682818 decreased the expression of mature miR-618 in 293T cells (Fig. 1f).

### miR-618 suppresses malignant phenotypes of CRC cell lines in vitro

**Figure 2:**
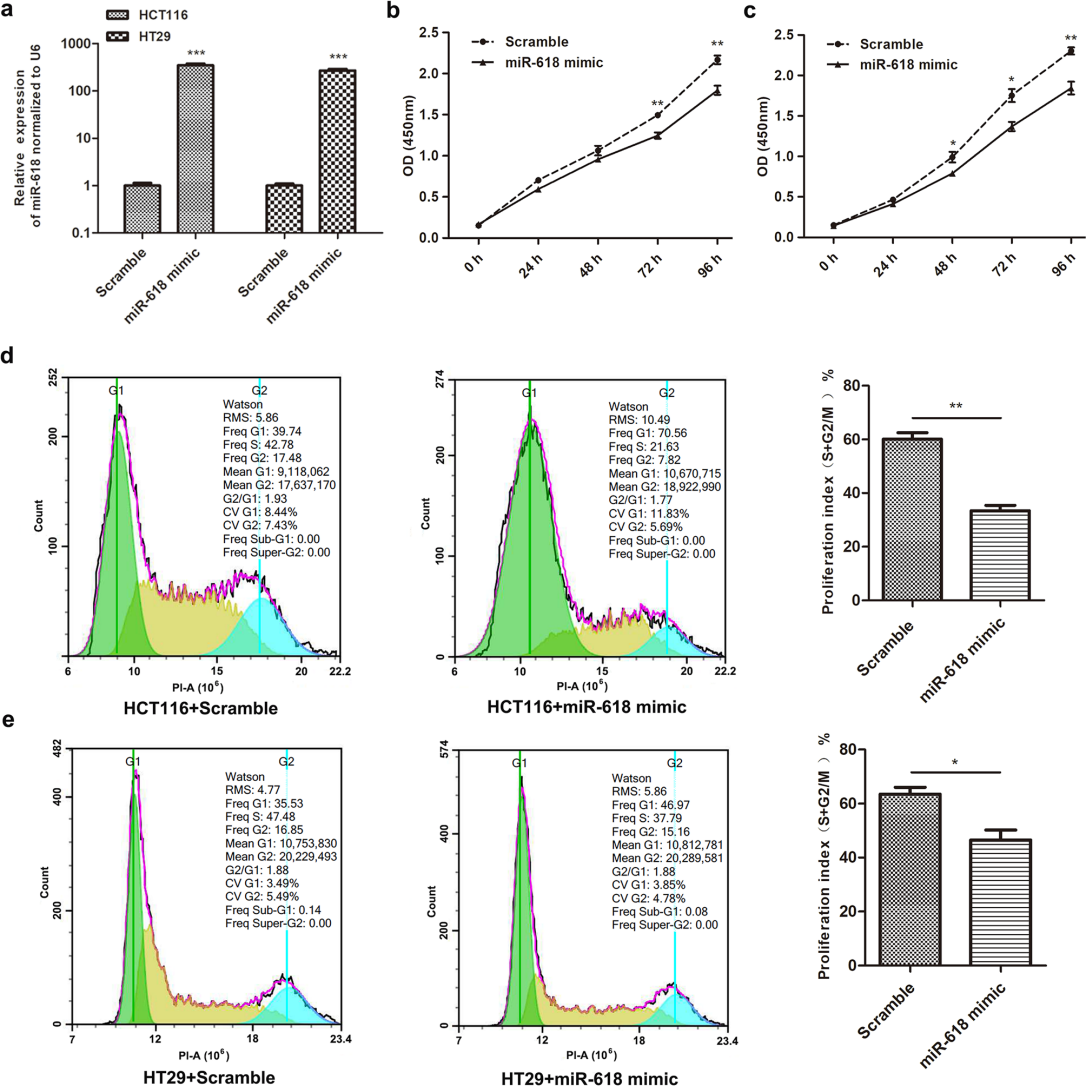
miR-618 suppresses the proliferation of colon cancer cell lines. (**a**) q-PCR analysis of miR-618 in HCT116 and HT29 cells after transfecting miR-618 mimic or Scramble. ***P < 0.001 (t-test). (**b**) Growth curves of HCT116 cells transfected with miR-618 mimic or Scramble. **P < 0.01 (t-test). (**c**) Growth curves of HT29 cells transfected with miR-618 mimic or Scramble. *P < 0.05, **P < 0.01 (t-test). (**d**) Flow cytometry analysis of cell cycle of HCT116 cells transfected with miR-618 mimic or Scramble. **P < 0.01 (t-test). (**e**) Flow cytometry analysis of cell cycle of HT29 cells transfected with miR-618 mimic or Scramble. *P < 0.05 (t-test).

To furtherly clarify the association of Polymorphism rs2682818 with CRC risk, we assessed the biological function of miR-618 in CRC cell lines. As shown in Fig. 2a, miR-618 mimic was transfected into HCT116 and HT29 cells to increase the expression of miR-618. CCK8 showed that miR-618 mimic significantly suppressed the proliferation of HCT116 and HT29 cells (Fig. 2b,c), flow cytometry analysis also confirmed this phenomenon, the proliferation index of HCT116 and HT29 cells was reduced after miR-618 mimic transfection (Fig. 2d,e).

**Figure 3:**
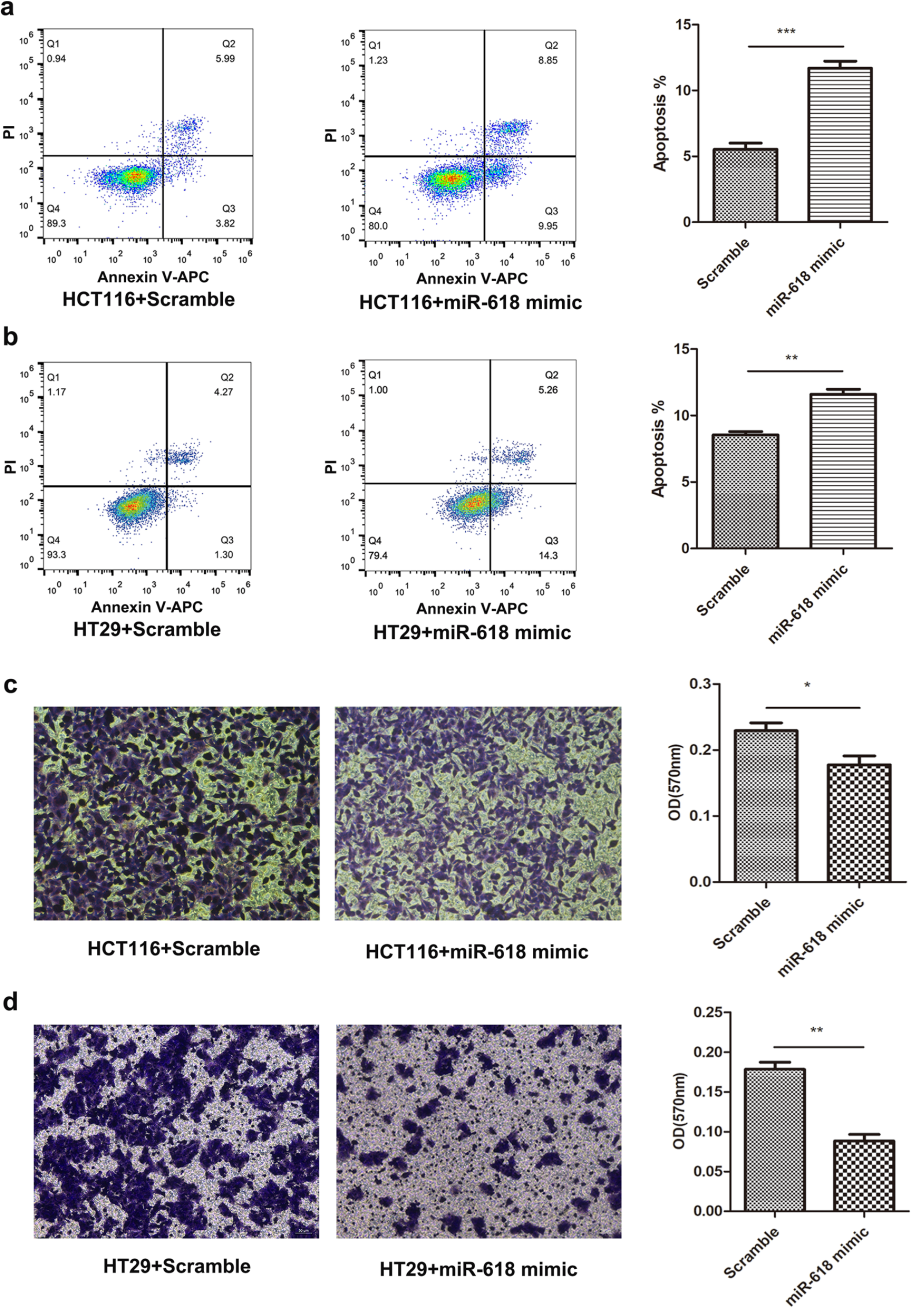
miR-618 inhibits the progression of colon cancer cell lines. (**a**) Flow cytometry analysis of apoptosis of HCT116 cells transfected with miR-618 mimic or Scramble. ***P < 0.001 (t-test). (**b**) Flow cytometry analysis of apoptosis of HT29 cells transfected with miR-618 mimic or Scramble. ***P < 0.001 (t-test). (**c**) Invasion assay of HCT116 cells transfected with miR-618 mimic or Scramble. *P < 0.05 (t-test). (**d**) Invasion assay of HT29 cells transfected with miR-618 mimic or Scramble. **P < 0.01 (t-test).

In addition, we analyzed the apoptosis rate of HCT116 and HT29 cells, our result showed that miR-618 notably promoted HCT116 and HT29 cells apoptosis (Fig. 3a,b). Furthermore, we also verified that the invasive ability of HCT116 and HT29 cells was significantly suppressed after miR-618 mimic transfection (Fig. 3c,d).

### miR-618 directly targets TIMP1 expression in CRC cells

**Figure 4:**
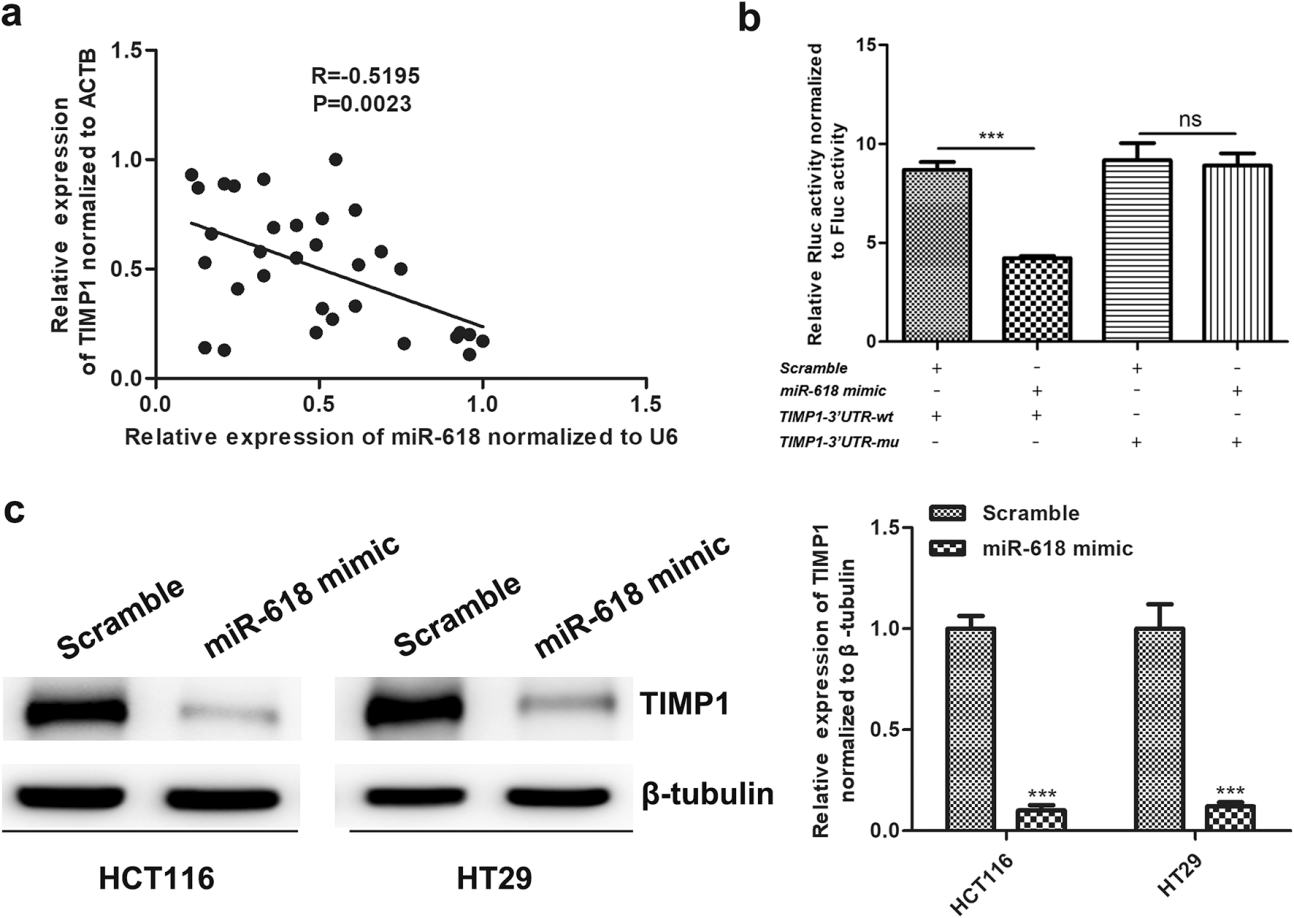
miR-618 directly targets TIMP1 expression in colorectal cancer. (**a**) The expression correlation analysis between miR-618 and TIMP1 in gastric colorectal cancer tissues (n = 32). R =  − 0.5195, P = 0.0023. (**b**) Luciferase reporter assay of HEK293T cells which were cotransfected with wild-type or mutant psiCHECK2-TIMP1-3'UTR and miR-618 mimics or Scramble. ns = no significance, ***P < 0.001 (t-test). (**c**) Western blot analysis of the TIMP1 in HCT116 and HT29 cells transfected with miR-618 mimic or Scramble (left), and the followed grayscale value analysis is shown (right). Data are reported as means ± SD of three independent experiments. **P < 0.01, ***P < 0.001 (t-test).

By bioinformatics prediction, we initially selected TIMP1, XIAP and TGFB2 as the candidate target genes of miR-618. And then, qPCR analysis was performed to confirm the real target gene of miR-618 in colorectal cells, and our result revealed that TIMP1 was the only one which was inhibited by miR-618 mimics in colorectal cells (data was not shown). Studies have proved that Tissue inhibitor matrix metalloproteinase 1 (TIMP1) plays a vital role in carcinogenesis^26^. And Pearson's correlation analysis revealed that there was a negative correlation between miR-618 and TIMP1 expression in CRC tissues (Fig. 4a). So, we further confirmed the target relationship of miR-618 and TIMP1 via luciferase assay (Fig. 4b) and western blotting (Fig. 4c).

### miR-618 inhibits CRC progression via inhibiting expression of TIMP1

**Figure 5:**
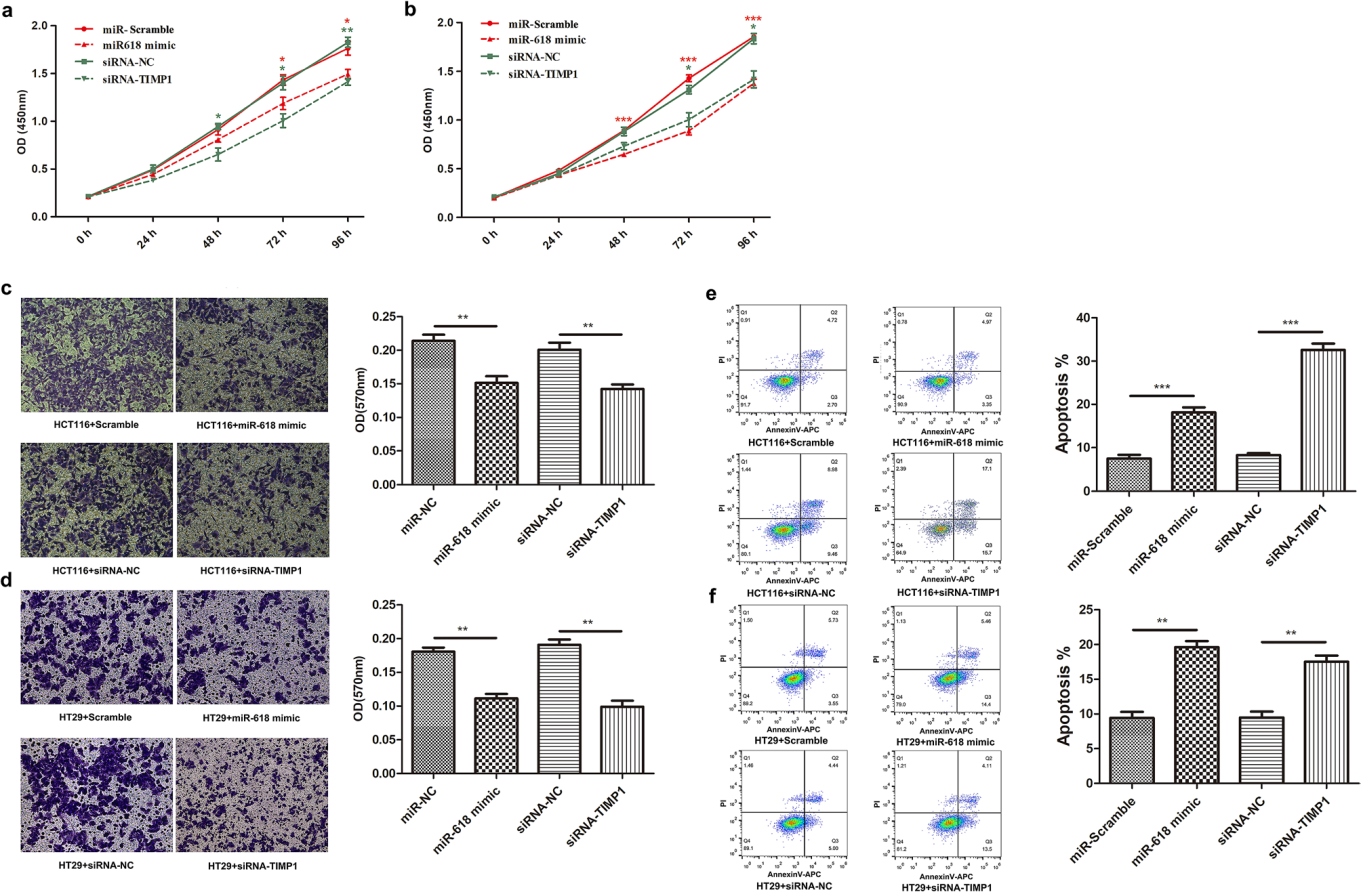
miR-618 and siRNA induced down-regulation of TIMP1 suppresses malignant phenotypes of HCT116 and HT29 cells. Growth curves of HCT116 (**a**) and HT29 (**b**) transfected with miR-618 mimic, miR-Scramble, siRNA-NC or siRNA-TIMP1were shown. The significance of the difference between group miR-Scramble and group miR-618 is indicated in red, as the significance of the difference between group siRNA-NC and siRNA-TIMP1 is indicated in green. *P < 0.05, **P < 0.01, ***P < 0.001 (t-test). (**c**) Invasion assay of HCT116 cells transfected with miR-618 mimic, miR-Scramble, siRNA-NC or siRNA-TIMP1. **P < 0.01 (t-test). (**d**) Invasion assay of HT29 cells transfected with miR-618 mimic, miR-Scramble, siRNA-NC or siRNA-TIMP1. **P < 0.01 (t-test). (**e**) Flow cytometry analysis of apoptosis of HCT116 cells transfected with miR-618 mimic, miR-Scramble, siRNA-NC or siRNA-TIMP1. ***P < 0.001 (t-test). (**f**) Flow cytometry analysis of apoptosis of HT29 cells transfected with miR-618 mimic, miR-Scramble, siRNA-NC or siRNA-TIMP1. **P < 0.01 (t-test).

**Figure 6:**
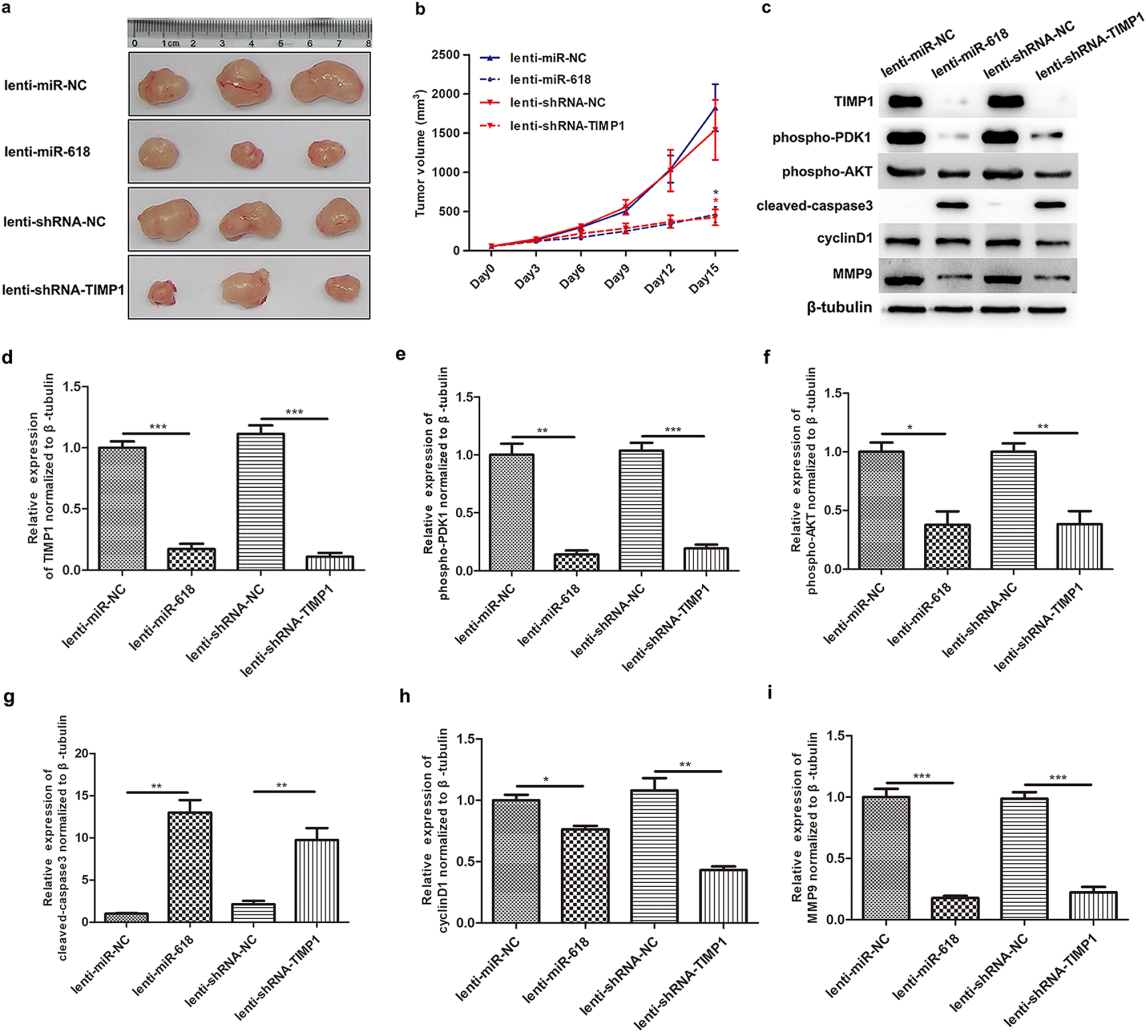
miR-618 suppresses the tumorigenesis of HT29 in vivo via inhibiting TIMP1 expression. (**a**) Representative images of tumors isolated from nude mice transplanted with HT29 subcutaneously. (**b**) Growth curves of tumors above-mentioned. miR-618 over-expression lenti-virus suppressed the growth of subcutaneous xenografts, and so did the TIMP1 shRNA lenti-virus. The significance of the difference between group lenti-miR-NC and group lenti-miR-618 is indicated in blue, as the significance of the difference between group lenti-shRNA-NC and lenti-shRNA-TIMP1 is indicated in red. *P < 0.05 (t-test). (**c**) Malignant phenotype related regulators were detected by western blot in tumors above-mentioned. (**d**–**i**) Followed grayscale value analysis of these regulators in tumors. Data are reported as means ± SD of three independent experiments, and statistical analyses are done for groups with lines, and the adjusted *p* value are represented above the lines. *P < 0.05,**P < 0.01, ***P < 0.001, and ns (not significant) with *P* > 0.05 (t-test).

In order to clarify the roles of miR-618-TIMP1 axis in CRC progression, we verified the effect of miR-618 mimics and TIMP1 siRNA on CRC cells in Parallel. CCK8 showed that TIMP1 siRNA significantly suppressed the proliferation of HCT116 (Fig. 5a) and HT29 cells (Fig. 5b). Furthermore, we also verified that the invasive ability of HCT116 (Fig. 5c) and HT29 cells (Fig. 5d) was significantly suppressed after TIMP1 siRNA transfection. Flow cytometry analysis also confirmed that TIMP1 siRNA remarkably promoted apoptosis of HCT116 (Fig. 5e) and HT29 (Fig. 5f) cells. All of above parallel studies revealed that miR-618 mimics played similar roles as TIMP1 siRNA in HCT116 and HT29 cells. In order to further verify the biological function of miR‐618/TIMP1 axis, we conducted an *in viv*o study, in which lentivirus were used to express TIMP1 siRNA and miR-618. Mouse xenograft assay indicated that over-expression of miR-618 could significantly suppress tumor growth. As the same, knock down of TIMP1 also could slowdown tumor growth in vivo (*P* < 0.05, Fig. 6a,b).

### miR-618-TIMP1 axis regulates CRC progression via PDK1/AKT signaling pathway

As reported in previous research, TIMP1 regulated progression in melanoma via PDK1 and downstream pathway^27^, we detected phosphorylated PDK1 (Ser 241) and phosphorylated AKT (Thr 308) using western blotting in neoplasm tissues dissected from nude mice. The results of western blotting presentation (Fig. 6c) and corresponding analysis (Fig. 6d–i) were shown respectively. In detail, PDK1 (Ser 241) phosphorylation was significantly suppressed by TIMP1 siRNA or miR-618 lentivirus. In addition, the expression of phosphorylated AKT (Thr 308) was also remarkably decreased after TIMP1 siRNA or miR-618 lentivirus infection. As the downstream apoptosis markers, cleaved-caspase3 was up-regulated after TIMP1 siRNA or miR-618 lentivirus infection, while cyclinD1 and MMP9 were decreased.

## Discussion

Colorectal carcinoma (CRC) is the third most common cancer and a significant cause of cancer-related deaths worldwide. Screening new susceptibility markers is of great significance for early prevention of CRC and reduction of mortality^28–30^. Previous studies revealed that single nucleotide polymorphisms (SNPs) were associated with various cancers risk and progression, including SNPs in microRNAs (miR-SNPs)^16–18^. SNP rs12402181 and rs62571442 in miR-3689d2 and miR-3117 were proved to be associated with acute lymphoblastic leukemia^31^. SNP rs67106263 in mir-3144 was associated with the carcinogenesis in thyroid tissue^32^. Notably, SNP rs11614913 in miR-196a2 was proved to be related to the high susceptibility of CRC^33–35^, and SNP rs2910164 in miR-146a was associated with the high risk of CRC in Chinese Han population^36^. However, the underlying molecular mechanisms of SNPs regulating CRC are still need to be explored.

SNP rs2682818 is loaded in the stem-loop sequence of pre-miR-618. Previous studies have shown that it is related to many diseases, including breast cancer, lymphoma and Ischemic Stroke^37–39^. Chen and colleagues have proved that SNP rs2682818 in pre-miR-618 is associated with CRC susceptibility in a Han Chinese population, however, the underlying mechanism is still unclear^25^. In this study, we further proved that SNP rs2682818 was associated with CRC progression and survival. And moreover, we found that the GG homozygous or TG heterozygous genotype of rs2682818 were associated with decreased expression of mature miR-618 compared with the TT homozygous genotype. This result suggested that SNP rs2682818 regulated the expression of mature miR-618.

Previous studies have demonstrated an association between aberrant miR‐618 expression and tumorigenesis, and miR‐618 may act as either an oncogene or a tumor suppressor in different types of cancer^40^. miR‐618 was deemed as a tumor suppressor in anaplastic thyroid cancer by targeting the XIAP gene^41^. And in another study, miR‐618 was proved to be a tumor suppressor in gastric cancer by downregulating the expression of TGF-β2^42^. However, miR‐618 was also reported to act as an oncogene in other cancers, including breast cancer and bladder cancer^43,44^. A study of head and neck squamous cell carcinoma revealed that miR‐618 participated in cancer progression by regulating the E2F1 and SMAD genes expression^45^. These above-mentioned findings suggest that the function of miR-618 may vary in diverse cancers. However, the precise roles and molecular mechanisms of miR‐618 in CRC are still unclear. In the study, we found that miR-618 suppressed many malignant phenotypes of CRC in vitro, such as malignant proliferation and high invasiveness. In addition, miR-618 could significantly promote CRC cell apoptosis. Our results suggested miR‐618 acted as a tumor suppressor in CRC.

To explore the underlying molecular mechanism of miR‐618 in CRC, we reviewed the databases of miRNAs, and we identified TIMP1 as a candidate downstream target of miR‐618. Tissue inhibitor matrix metalloproteinase 1 (TIMP1) belongs to the Tissue Inhibitor of Metalloproteinases family^16^. Previous studies have demonstrated that overexpression of TIMP1 can promote cell proliferation and inhibit cell apoptosis in a variety of cancers^46^. Specifically, the TIMP1 can degrade cyclinB1 and activate the NF-κB signaling pathway to protect breast cancer cells against chemotherapy-induced cell death^47^. In addition, it has also been proved that TIMP1 might exert anti-tumor effect by binding to the CD63/integrin β1 complex^48^. In this study, we well confirmed that miR‐618 targeted TIMP1 in CRC cells by western blotting and luciferase reporter assay. Furtherly, we verified the effects of TIMP1 in CRC cells independently, and our results revealed that TIMP1 acted as an oncogene in CRC, as it could promote CRC cell proliferation and metastasis.

Previous studies had proved that there were different downstream signaling pathways involved in the effect of TIMP1^26,27^. Mariana Toricelli and colleagues had reported that TIMP1 promoted cell survival by activating the PDK1 signaling pathway in melanoma^27^. Gagliardi PA and colleagues had proved the PDK1 played a critical role in cell proliferation and apoptosis through AKT signaling pathway^49^. Besides, Dai W had revealed that PDK1 deficiency blocked proliferation of colon cancer cells and led to apoptosis^50^. Based on these above evidences, we repeatedly verified this signaling pathway in xenograft tissues of HT29, our results confirmed that PDK1-AKT indeed involved in the progression of HT29 xenograft as the downstream signaling of TIMP1.

Although our study have provided evidence that SNP rs2682818 is involved in CRC progression by affecting the expression of miR-618, and it also have proved that miR-618 regulate the progression of CRC via modulating the expression level of TIMP1, the signal pathways downstream of miR-618-TIMP1 signal axis are still not very clear, we will continue to explore the deeper molecular mechanisms.

## Methods

### Patients and tissue specimens

The present study was approved by the Ethics Committee of Zhoushan Putuo District People's Hospital (Zhoushan, Zhejiang Province). All of the 205 patients with colorectal cancer and 39 patients with colitis involved in this study provided written informed consent prior to sample collection. Among them, the fresh tumor and colitis tissue specimens were collected during surgical resection at Zhoushan Putuo District People's Hospital between January 2011 and December 2013. All tissue specimens were confirmed by pathological analysis and immediately put into liquid nitrogen until DNA and RNA extraction. The corresponding patient characteristics were collected and the 5-year survival information was also collected and recorded for subsequent analysis. The SNP analysis of all these specimens were provided by BGI Genomics Co., Ltd (Shenzhen, Guangdong Province). All methods were performed in accordance with the relevant guidelines and regulations. Informed consent was obtained from all participants.

### Plasmid construction

miR-168 precursor sequences harboring rs2682818 and without rs2682818 were obtained by de novo synthesis, which were then separately digested with BamH I and Xho I (New England Biolabs, Ipswich, MA, USA) and cloned into the pcDNA6.2-GW/EmGFP plasmid (Invitrogen, Karlsruhe, Germany). The Wild type 3’UTR sequence of TIMP1 (TIMP1-3’UTR-wt) and 3’UTR sequence of TIMP1 without predicted miR-168 target site (TIMP1-3’UTR-mu) were obtained by de novo synthesis, which were then separately digested with Xho I and EcoR I(New England Biolabs, Ipswich, MA, USA) and constructed into psiCHECK2 plasmid (Promega, Madison, WI, USA) especially.

### Luciferase reporter assay

293T cells (10^5^ Cells) were seeded in 24-well plates before transfection. According to the instructions, miR-618 mimics and TIMP1 3’UTR Reporter plasmids (psiCHECK-TIMP1-3’UTR-wt or psiCHECK-TIMP1-3’UTR-mu) were co-transfected using Lipofectamine 2000 reagent (Invitrogen, Karlsruhe, Germany) with final concentration of 50 nM(mimics) and 200 ng(3’UTR Reporter plasmid). And 48 h later, 293T cells were collected and Luciferase activity was detected using the Dual-Glo luciferase assay system (Promega, Madison, WI, USA) according to the manufacture’s protocol.

### Cell culture and transfection

The colorectal carcinoma cell lines HT29, HCT116, Lovo, SW480, SW620 were purchased from Cell bank of Chinese Academy of Sciences. These cells were maintained in McCoy's 5a (HT29 and HCT116) or L15 (SW480 and SW620) and F12K (Lovo) medium supplemented with 10% fetal bovine serum (Hyclone, Salt Lake City, UT, USA), 100 units/ml penicillin and 100 units/ml streptomycin (sigma, St-Louis, MO, USA). All these cells were cultured in a humidified environment containing 5% CO2 and held at a constant temperature of 37 °C.

TIMP1 siRNA, negative control (NC) siRNA, miR-618 mimics and scramble mimics were all obtained from Gene-Pharma (Shanghai, China). All of siRNAs, mimics in this study were transfected with Lipofectamine 2000 reagent (Invitrogen, Karlsruhe, Germany) according to the manufacturer’s instructions. Cells were seeded in 6-well plates at a concentration of 2*10^5^ cells/well, and were transfected with siRNA or mimics when cells reached 40–60% confluence.

For verification of the expression efficiency, the two different miR-168 plasmids were separately transfected into 293T cells. In detail, cells were seeded at 50% confluency in six-well and cultured overnight. Plasmids (10 µg per-well for six-well) were transfected with Lipofectamine 2000 reagent (Invitrogen, Karlsruhe, Germany) according to the manufacturer’s instructions.

### q-PCR

Total RNA was extracted from tissue specimens and cultured cells with TRIzol reagent (Invitrogen; Thermo Fisher Scientific, Inc.). The total RNA concentration and quality of each sample were assessed using a NanoDrop one spectrophotometer (NanoDrop Technologies; Wilmington, DE, USA) and gel electrophoresis. The RNA with 260/280 ratio between 1.9 and 2.1, 260/230 ratio greater than 2.0, and clear bands for 28S and 18S after running the RNA on an agarose gel were used for cDNA Synthesis. For TIMP1 expression analysis, cDNA was generated from 1 µg of total RNA using the PrimeScript RT Reagent Kit, and then the synthesized cDNA was diluted at 1:10 as the templates. q-PCR was performed using SYBR Premix Ex Taq (Takara Biotech, Dalian, China) according to the manufacturer’s manual, and the level of glyceraldehyde-3-phosphate dehydrogenase (GAPDH) was used as a control. For miR-618 expression analysis, cDNA was generated from 1 µg of total RNA using the miRNeasy mini kit (QIAGEN, Hilden, Germany) according to the manufacturer’s manual, and then the synthesized cDNA was diluted at 1:10 as the templates. Especially, the q-PCR reactions of miR-618 and U6 were performed according to the manufacturer’s instructions of All-in-One™ miRNA q-PCR Detection Kit (GeneCopoeia, Rockville, MD, USA), and among them, snRNA U6 was used as a control. iQ-5 (Bio-Rad, Hercules, CA, USA) was used to monitor all these q-PCR reactions, an *R*^2^ ≥ 0.9999 and the efficiency (*E*) = 100 ± 5% were required for the best amplification of each gene. RNA expression was relative quantified using 2^−ΔΔCt^ method.

### Western blotting

Total protein was extracted from cell lines and tissues using RIPA protein lysis buffer (Beyotime, shanghai, china) with added 1% protease inhibitor cocktail and 1 mM phenylmethylsulfonyl fluoride (PMSF), and the protein concentration was measured using BCA Protein Assay kit (Beyotime). Generally, 50 μg of protein was used for western blotting. The obtained proteins were then separated by SDS-PAGE and transferred onto a 0.45 μm cellulose acetate membrane (Millipore, MA, USA). After blocking in 5% skim milk, the PVDF membranes were incubated with primary antibodies in blocking buffer overnight at 4 °C and then with HRP-conjugated secondary antibody for 2 h. The primary antibodies used were: anti-β-tubulin (1:5000 dilution, Santa Cruz, CA, USA), anti-TIMP1 (1:1000 dilution, Abcam, Cambridge, UK), anti- phospho-PDK1 (1:1000 dilution, CST, MA, USA), anti- phospho-AKT (1:1000 dilution, CST), anti- cyclinD1 (1:1000 dilution, Abcam), anti-MMP9 (1:1000 dilution, Abcam), anti-cleaved caspase3 (1:1000 dilution, Abcam). Reactive bands were visualized with ECL reagent (Pierce, Rockford, IL) and analyzed. Protein expression was quantified using ImageJ software (National Institutes of Health, Bethesda, MD, USA).

### Cell viability assay

HT29 and HCT116 Cells were seeded respectively in 96-well plates in triplicate at densities of 5000 cells per well. Cell viability was evaluated at desired time points using CCK8 kits (Dojindo Molecular Technologies, Kumamoto, Japan) according to the instructions. Light absorbance of the solution was measured at 450 nm on a microplate reader.

### Cell invasion assay

Transwell chambers coated with Matrigel (BD Biosciences, NJ, USA) were used to analysis cell invasion. HT29 and HCT116 Cells in 100 μl serum-free McCoy's 5a medium were seeded on upper chambers in triplicate at densities of 2*10^5^ and McCoy's 5a with 10% FBS was added to lower chambers. After 24 h incubation, invaded cells in the lower side of the membranes were fixed with methanol and stained with Crystal Violet. Images were taken using an inverted microscope. Invaded cells were counted from three different fields. The experiment was repeated three times (Supplementary file S1).

### Apoptosis assay

Cells were harvested, washed, and resuspended in 1 ml of binding buffer, and then stained with 5 μl of fluorescein isothiocyanate–Annexin V (BD Biosciences, San Jose, CA) and 10 μl of propidium iodide (Sigma-Aldrich) in the dark for 15 min at room temperature. At last, these cells were analyzed by flow cytometry (BD Biosciences) equipped with a CellQuest software.

### Xenograft mouse model

BABL/c nude mice were obtained from shanghai SLAC laboratory animal Co., Ltd. The animals were administered a standard rodent diet with free access to water (ad libitum) and were housed in rooms sustained at 22 ± 1 °C with a 12 h light/dark cycle. The experimental protocol was reviewed and approved by the Animal Ethics Committee of zhejiang University. Generally, each nude mice were injected with 100uL of HT29 cells subcutaneously according to the preimplantation experiment, When the tumor volume up to 100 mm^3^, mice were randomly divided into four groups (3 mice per group) to accept different lentivirus intervention, including lenti-miR-NC, lenti-miR-618, lenti-shRNA-NC and lenti-shRNA-TIMP1, virus were injected intratumoral once every three days with a dose of 10^7^ pfu per mouse, and the size of the tumor was measured. Two weeks later, the subcutaneous tumors were stripped. Tumor volume was calculated according to the formula: V (mm^3^) = 0.5 * a * b^2^ (a represents the longest axis and b the shortest axis). Lentil-virus used in this study were kindly provided by shanghai R&S biotechnology Co., Ltd (shanghai, china).

### Bioinformatics and statistical analysis

Target genes of miR-618 were predicted by MIRDB (http://mirdb.org/), TargetScan 5.1 (http://www.targetscan.org/), and miRanda (http://www.microrna.org/). Results are presented as means ± standard deviations of three independent experiments. Significant differences in the mean values were evaluated by unpaired t-test. One-way ANOVA was used to compare continuous variables among two or more groups. Tests of association were conducted using Pearson's χ^2^ test. p < 0.05 was considered statistically significant.

### Ethics approval and consent to participate

The experimental protocol was reviewed and approved by the Animal Ethics Committee of Zhejiang University.

## Supplementary Information

Supplementary Information.

## Data Availability

All data generated or analyzed during this study are included in this article.
