# Supplementary material for: Polymorphism rs2682818 participates in the progression of colorectal carcinoma via miR-618-TIMP1 regulatory axis
Source: Sci Rep. 2021 Nov 30;11:23186. doi: 10.1038/s41598-021-02613-4 (PMC8632919; doi:10.1038/s41598-021-02613-4)
Supplement: Supplementary file 1 — Supplementary Information. [file 41598_2021_2613_MOESM1_ESM.pdf]

Supplementary materials

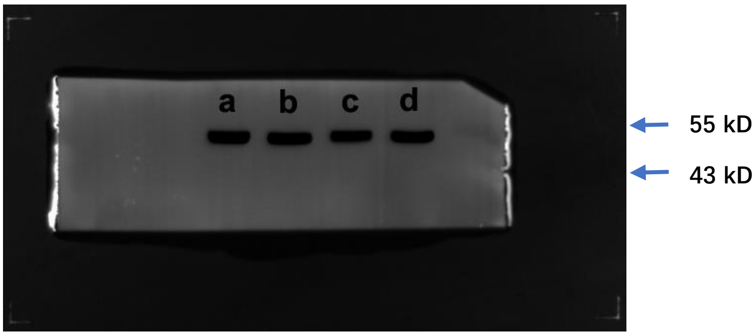

Supplementary figure 1. The original image of western blot of β-tubulin in Figure 4. a, The scramble transfected HCT116 cells. b, miR-618 mimics transfected HCT116 cells. c, The scramble transfected HT29 cells. d, miR-618 mimics transfected HT29 cells. The predicted band size: 50 kDa.

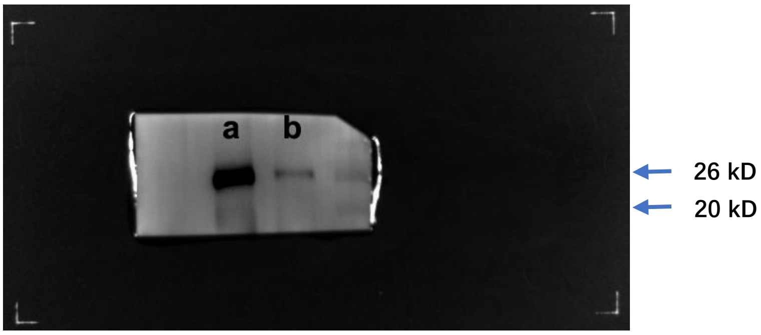

Supplementary figure 2 The original image of western blot of timp1 in Figure 4. a, The scramble transfected HCT116 cells. b, miR-618 mimics transfected HCT116 cells. The predicted band size: 23 kDa.

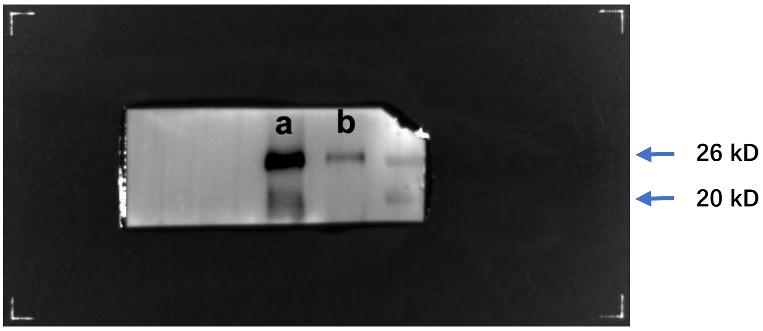

Supplementary figure 3 The original image of western blot of timp1 in Figure 4. a, The scramble transfected HT29 cells. b, miR-618 mimics transfected HT29 cells. The predicted band size: 23 kDa.

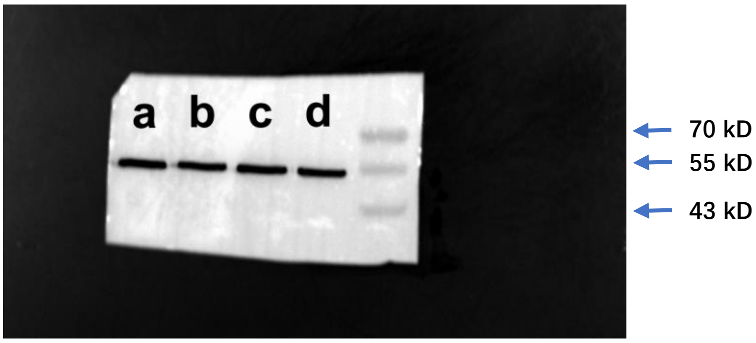

Supplementary figure 4. The original image of western blot of β-tubulin in Figure 6. a, The miR-NC lenti-virus infected xenograft of HT29 cells. b, The miR-618 lenti-virus infected xenograft. c, The shRNA-NC lenti-virus infected xenograft. d, The shRNA-TIMP1 lenti-virus infected xenograft. The predicted band size: 50 kDa.

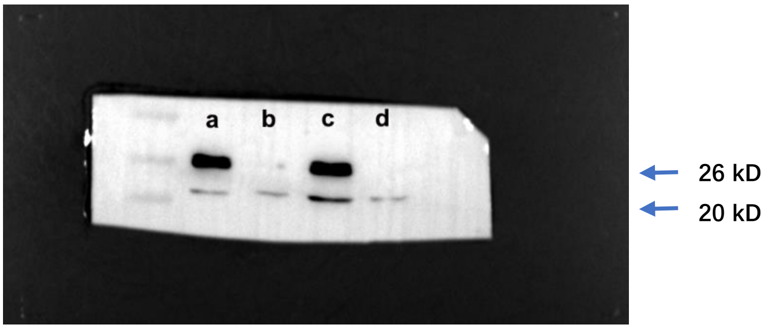

Supplementary figure 5. The original image of western blot of TIMP1 in Figure 6. a, The miR-NC lenti-virus infected xenograft of HT29 cells. b, The miR-618 lenti-virus infected xenograft. c, The shRNA-NC lenti-virus infected xenograft. d, The shRNA-TIMP1 lenti-virus infected xenograft. The predicted band size: 23 kDa.

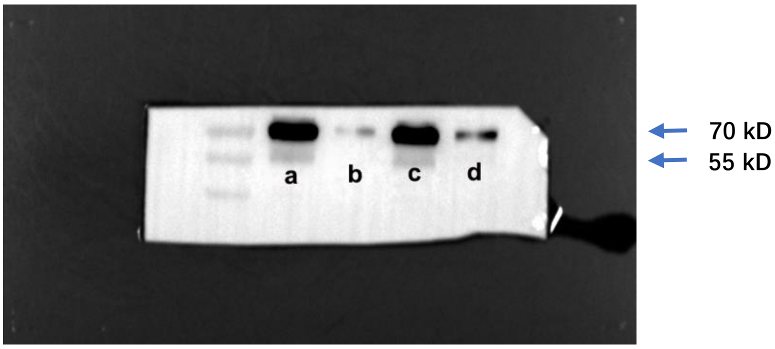

Supplementary figure 6. The original image of western blot of phospho-PDK1 in Figure 6. a, The miR-NC lenti-virus infected xenograft of HT29 cells. b, The miR-618 lenti-virus infected xenograft. c, The shRNA-NC lenti-virus infected xenograft. d, The shRNA-TIMP1 lenti-virus infected xenograft. The predicted band size: 58-68 kDa.

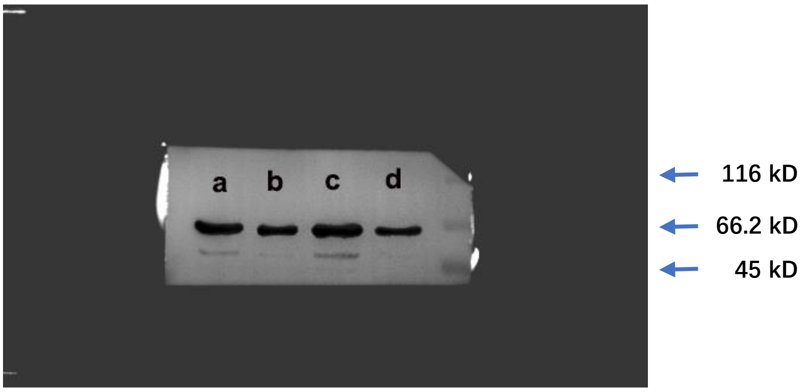

Supplementary figure 7. The original image of western blot of phospho-AKT in Figure 6. a, The miR-NC lenti-virus infected xenograft of HT29 cells. b, The miR-618 lenti-virus infected xenograft. c, The shRNA-NC lenti-virus infected xenograft. d, The shRNA-TIMP1 lenti-virus infected xenograft. The predicted band size: 60 kDa.

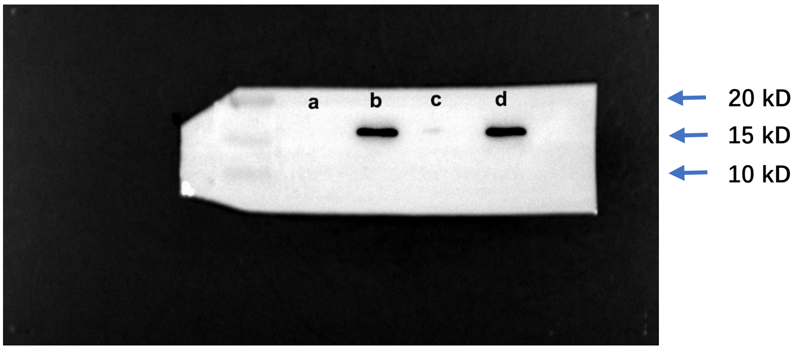

Supplementary figure 8. The original image of western blot of cleaved caspase3 in Figure 6. a, The miR-NC lenti-virus infected xenograft of HT29 cells. b, The miR-618 lenti-virus infected xenograft. c, The shRNA-NC lenti-virus infected xenograft. d, The shRNA-TIMP1 lenti-virus infected xenograft. The predicted band size: 17 kDa.

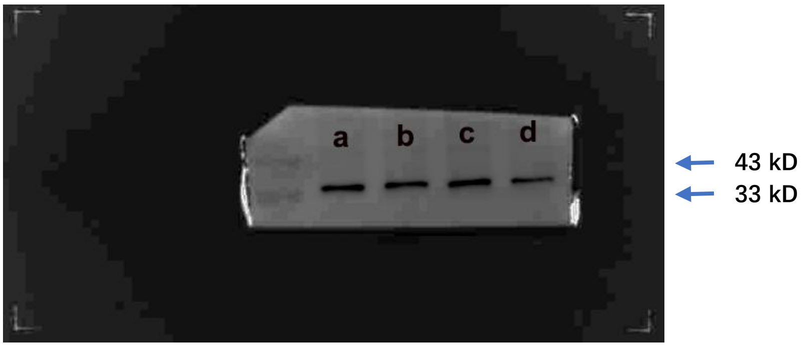

Supplementary figure 9. The original image of western blot of cyclinD1 in Figure 6. a, The miR-NC lenti-virus infected xenograft of HT29 cells. b, The miR-618 lenti-virus infected xenograft. c, The shRNA-NC lenti-virus infected xenograft. d, The shRNA-TIMP1 lenti-virus infected xenograft. The predicted band size: 33 kDa.

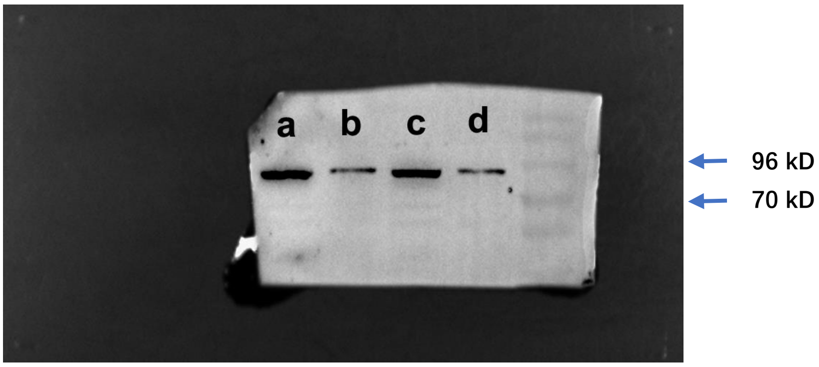

Supplementary figure 10. The original image of western blot of MMP9 in Figure 6. a, The miR-NC lenti-virus infected xenograft of HT29 cells. b, The miR-618 lenti-virus infected xenograft. c, The shRNA-NC lenti-virus infected xenograft. d, The shRNA-TIMP1 lenti-virus infected xenograft. The predicted band size: 78 kDa.
